# Exploring the practicality and acceptability of a brief exercise communication and clinician referral pathway in cancer care: a feasibility study

**DOI:** 10.1186/s12913-023-10003-x

**Published:** 2023-09-22

**Authors:** Cristina M. Caperchione, Madeleine English, Paul Sharp, Meera R. Agar, Jane L. Phillips, Winston Liauw, Carole A. Harris, Susan McCullough, Ruth Lilian

**Affiliations:** 1 School of Sport, Exercise and Rehabilitation, University of Technology Sydney, Sydney, NSW Australia; 2 IMPACCT, University of Technology Sydney, Sydney, NSW Australia; 3 School of Nursing, Faculty of Health, Queensland University of Technology, Brisbane, QLD Australia; 4 Cancer Care Centre, St George Hospital, Sydney, NSW Australia; 5 St George and Sutherland Clinical School, University of New South Wales, Sydney, NSW Australia; 6 Translational Cancer Research Network, Sydney, NSW Australia

**Keywords:** Cancer and exercise, Cancer care services, Exercise referral, Oncologist-patient communication, Exercise physiologist, Exercise professionals, Integrated clinical practice

## Abstract

**Background:**

The majority of cancer patients and cancer care clinicians-CCCs (e.g., oncologists) believe that exercise is an important adjunct therapy that should be embedded in standard practice. Yet, CCCs do not routinely discuss exercise with their patients, nor do they regularly refer them to exercise professionals (e.g., exercise physiologists-EPs). This study evaluated the feasibility and acceptability of an evidence-based approach to improving exercise communication between CCCs and their patients, including an exercise referral pathway.

**Methods:**

Implementation and testing of the Exercise Communication and Referral Pathway (ECRP) occurred in Sydney, Australia. The ECRP included a brief oncology-initiated communication exchange with patients, CCC exercise referral to an EP, followed by EP-initiated telephone consultation with patients concerning tailored exercise advice. Participant perceptions concerning the feasibility and applicability of the ECPR were evaluated. Semi-structured interviews were conducted with CCCs (n = 3), cancer patients (n = 21), and an EP (n = 1). Inductive thematic analysis was undertaken.

**Results:**

Analysis generated three themes: (1) Navigating the role of CCCs in the ECRP, suggesting that oncology-initiated communication is a cue to action, however there was a lack of role clarity regarding exercise referral; (2) Implementing Patient-Orientated Care within a Standardised Pathway, highlighting the need for tailored information and advice for patients that reflects individual disease, socio-cultural, and environmental factors, and; (3) Taking Steps Towards Action, revealing the need for structural (e.g., EP initiated contact with patients) and policy changes (i.e., changes to Medicare, direct oncologist referral) to engage patients and better integrate exercise as part of standard care.

**Conclusions:**

Findings provide important insights into improving oncology-patient exercise communication and developing an exercise referral pathway to increase engagement and patient reach. However, individual (e.g., experience, knowledge) and contextual factors (e.g., time, resources) need consideration when implementing an ECRP.

**Trial registration:**

This trial was prospectively registered with the Australian New Zealand Clinical (#ACTRN12620000358943) on March 13, 2020.

**Supplementary Information:**

The online version contains supplementary material available at 10.1186/s12913-023-10003-x.

## Background

Exercise is a safe and effective intervention that may counteract adverse physical and psychological effects of cancer and its treatment, including reducing pain and fatigue, and improving quality of life [1–4]. There is also evidence that regular exercise may be protective against cancer recurrence, cancer-specific mortality, and all-cause mortality for some types of cancer (e.g., breast, colorectal, prostate cancer) [2, 5, 6]. Despite these benefits and the burgeoning clinical support for promoting exercise as a standard of cancer care [1, 7], exercise is not routinely discussed during clinical consultations [8–10] and exercise professionals are not regularly integrated into clinical care [11, 12].

Efforts are needed to increase the proportion of people with cancer who meet recommended physical activity guidelines (i.e., 150 min moderate intensity or 75 min of vigorous intensity aerobic exercise and two to three strength training sessions per week [13]) [14, 15]. While only 17–47% (varies by tumour site) of people with cancer meet recommended exercise guidelines [16–18], surveys reveal the vast majority have positive perceptions of exercise during cancer treatment [19]. Similarly, the cancer care workforce have favourable attitudes [8, 12, 20], and believe that exercise is an important adjunct therapy and should be embedded as part of standard practice [7, 21, 22]. There are significant barriers to the implementation of exercise advice and referral as a standard of care in oncology [11, 12, 14], including insufficient time, uncertainty of what to recommend, and a lack of knowledge and confidence to discussing and promoting exercise with patients [8, 11, 12]. Additionally, clinical settings often present unique contextual and structural barriers to promoting exercise related to patient flow, clinician training, patient funding schemes, and resource availability (e.g., exercise professionals, space, equipment) [11, 12]. Addressing these barriers requires clear processes and procedures to help facilitate exercise communication from cancer care clinicians (CCCs) and support patients to engage in regular exercise throughout their cancer trajectory.

In Australia, the Clinical Oncology Society of Australia (COSA) provides best practice guidelines for health professionals whose work encompasses cancer control and care. Exercise related guidelines from COSA encourages CCCs (and other health professionals) to (1) discuss the role of exercise in cancer recovery with patients; (2) recommend to their patients to adhere to the exercise guidelines; and (3) refer their patients to a professional who specialises in the prescription and delivery of exercise (i.e., accredited EPs or physiotherapist with experience in cancer care) [4]. Nonetheless, many patients are unaware of these specialty EP services, how to access them, and their relevant financial support schemes. Further, CCCs exercise communication and referrals for their patients remains low, and thus, effective utilisation of EPs is at a minimum [10]. Within Medicare (Australia’s national health funding structure) general practitioners-GPs (i.e., family physicians) can refer patients with complex conditions (e.g., diabetes, cardiovascular disease, cancer, etc.) to a care plan, providing patients with a rebate for five allied health services/sessions (e.g., EP, nutritionist, psychologist). No other health professional, including oncologists, can establish a care plan to obtain these fully subsidised sessions. Patients can also access EP services privately without a referral, however, rebates through private health insurance companies are limited.

This lack of awareness and poor utilisation of EP services within the Australian healthcare system, could be overcome by better integrating EPs and other cancer specific exercise professionals into the cancer care team, allowing bidirectional gains in both knowledge of the oncologist in exercise oncology and the EP knowledge of the particular patient diagnosis and needs. There has been a global ‘call for action’ regarding this integration [11, 23], however, little has progressed across cancer care services despite evidence for exercise as part of standard oncology care [23]. In response to this call to action, the purpose of this study was to evaluate the feasibility and acceptability of an evidence-based approach to improving exercise communication between CCCs and their patients, inclusive of an exercise referral pathway from clinician to EPs.

## Methods

This study employed a qualitative descriptive design [24, 25] utilising semi-structured interviews to assess the feasibility and acceptability of a pragmatic Exercise Communication and Referral Pathway (ECRP) approach. Ethical approval for this study was provided by University of Technology Sydney Human Research Ethics Committee (#ETH18-3183) and the South Eastern and South Western Sydney Local Health District Human Research Ethics Committees (##2019/ETH00221). Methodological procedures and processes adhered to the Consolidated Criteria for Reporting Qualitative Research [26] (Related File 1).

### Exercise communication and referral pathway approach

Building on previous research and outcomes from our formative evaluation [12], a pragmatic approach to exercise communication and referral was developed in collaboration with CCCs and cancer patients (Additional File 1). CCCs were provided with an informational resource and 3-step guide (i.e., Assess, Aware, Advise) to engage in a brief (1–2 min) conversation about exercise with their cancer patients during a regular consultation or appointment. The structured conversation included targeted questions about the patient’s past and current exercise (i.e., pre/post diagnosis), information about the health benefits of exercise specific to that patient and the cancer they are living with, and the impact that exercise may have on treatment side-effects and long-term survivorship. Further, information about exercise recommendations for cancer patients [4] and different types of exercises that may be beneficial (e.g., walking, swimming, strength training) was shared with patients. CCCs were encouraged to tailor the conversation to their patients’ needs and interests with consideration to individual treatment factors (e.g., cancer type, stage, symptoms, etc.). To conclude, CCCs referred patients to a designated EP (accredited exercise physiologist with a PhD in Exercise Oncology) and indicated that the EP would contact them directly to further discuss their exercise and referral options (i.e., Medicare rebate). Within 2 weeks of the appointment with their CCC, patients were contacted by an EP via telephone. EP counselling lasted approximately 15–20 min and included: a brief medical and physical activity history, personalised physical activity recommendations based on patients’ disease stage/type, preferences and interests, identification of local EP clinics and other local exercise opportunities, and education regarding Medicare rebated EP sessions.

### Participants and recruitment

Participants were CCCs (e.g., oncologists) and cancer patients/survivors (≥ 18 years) from a public hospital in Sydney, Australia. A convenience sample of CCCs (n = 3) were recruited via email invitation and word-of-mouth from research team members affiliated with the hospital. Eligible CCCs were employed as an oncologist (i.e., radio/medical/surgical) or cancer focused haematologist at the hospital and currently seeing patients living with cancer. CCCs who indicated interest were sent an information and consent form to review and return to a member of the research team prior to the start of the trial. CCCs who consented to participate distributed letters of invitation to their patients to participate in the study. The EP involved in this study was selected for their cancer specific knowledge and expertise. The research team limited utilisation to one EP to ensure intervention consistency and trial efficacy. Eligible patients were adults (18 + years), able to speak and read English, currently receiving cancer treatment (any stage), and under the care of a participating CCC. CCC’s randomly invited eligible patients to partake in the study, to promote a representative sample. Rolling recruitment was ceased once data saturation was reached for cancer patients (n = 21), acknowledging that it was unlikely that additional interviews would yield new information. All participants provided written informed consent prior to participating in the ECRP and the interview post ECRP. Participants were assigned a code upon their recruitment to ensure privacy and confidentiality. An interview was also conducted with the community EP (n = 1) who provided the exercise counselling to participants to explore their perspectives and experiences with the ECRP.

### Procedures

Implementation of the ECRP approach was planned to occur over a 30-day period however, COVID-19 disruptions lead to this extending over a 3-month period between May-July 2021. The ECRP trial was deemed complete after successful contact by the EP to patient to discuss exercise and referral options. At this time, semi-structured telephone interviews were conducted to answer the research questions, (1) *Is the exercise communication strategy between CCCs and their patients feasible to undertake within a clinical setting*, and (2) *Does the exercise ECRP approach meet the needs of CCC and their patients living with cancer?* A relaxed conversational format was used to initiate the interviews, where the interviewer provided the participant with information about themselves as a way to build rapport with participants. This was followed by more specific open-ended questions that explored participants’ experiences and perspectives regarding the feasibility (e.g., delivery, uptake, and compliance) and acceptability (e.g., satisfaction, engagement, confidence, and importance) of the ECRP approach (Additional File 2). All interviews were conducted within 2 weeks of completing the ECRP by a female research team member (ME, BSportExerSci. (Hons), BHumanSci. and current PhD candidate) trained in qualitative data collection methods. They lasted approximately 30–40 min, were audio recorded using a digital SonyTM recorder (ICD-PX333), and further supported by supplementary notes taken by the interviewer (ME). Interviews were transcribed verbatim and deidentified using participant IDs (e.g., EP, CCC 1 or Patient 27). Participants were provided an opportunity to review their own transcript and provide further explanation, or revisions.

### Data analysis

Inductive thematic analysis was undertaken (using NVIVO 12 software), whereby patterns in the data were identified and described to interpret and explain what was said in addressing the research questions [27]. A coding framework was inductively developed to reflect important ideas represented in the interviews. Examples of codes utilised include *‘Perceived role of CCC’* and *‘Future directions for ECRP’*. Two researchers (ME, PS) independently coded one transcript to verify the consistency of the framework, and resolved areas of disagreement and refined categories. Coded data were reviewed by the lead author (CMC), examining similarities and differences within and across the interviews to determine preliminary themes. Throughout this stage of the analysis process, a particular focus was placed on data source triangulation [28] and comparing how patients, EP’s, and CCC’s experiences converged or diverged to develop a comprehensive understanding of participants’ perspectives on the ECRP.

## Results

**Fig. 1:**
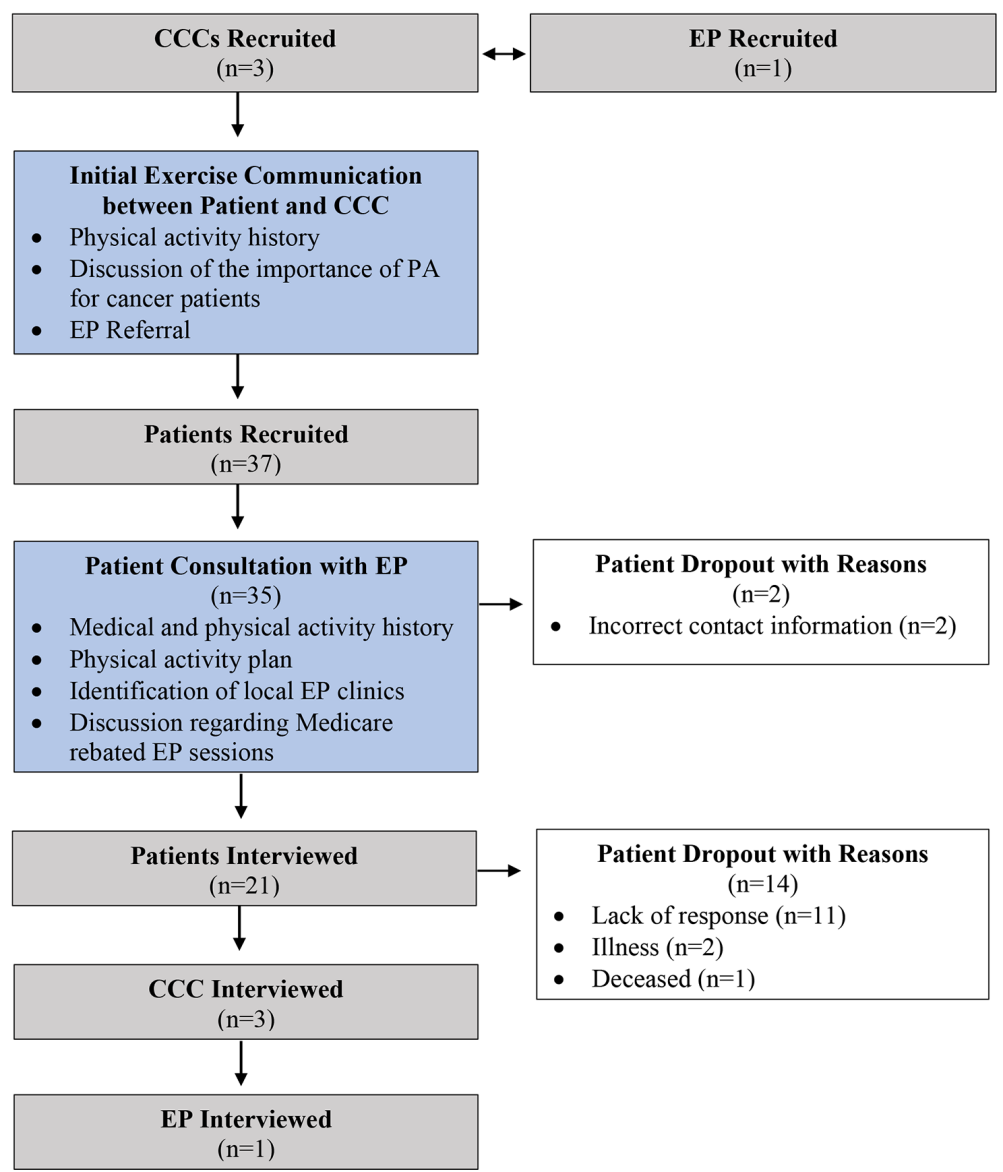
Overview of participant recruitment and flow

A total of 25 interviews were conducted, involving 21 out of the initially 37 recruited patients, 3 CCCs and the 1 EP. Figure 1 provides an overview of participant recruitment and flow. Table 1 outlines participant characteristics of the patients.

**Table 1:** Participant characteristics of cancer patients

*Variable*	*Participant Total %, (N = 21)*
Gender	
Male	38.1 (8)
Female	61.9 (13)
Cancer Stage	
Stage 1	14.3 (3)
Stage 2	4.8 (1)
Stage 3	14.3 (3)
Stage 4	19.0 (4)
Unknown	45.5 (10)
Cancer Diagnosis*	
Breast Cancer	45.5 (10)
Genitourinary Cancer	23.8 (5)
Lung Cancer	9.5 (2)
Bone Cancer	9.5 (2)
Brain Cancer	9.5 (2)
Gastrointestinal Cancer	9.5 (2)
Other	9.5 (2)

*NOTE: Some patients reported more than one cancer diagnosis

Data analysis from interviews generated three themes (1) *Navigating the role of Cancer Care Clinicians in the ECRP*, (2) *Implementing Patient-Orientated Care within a Standardised Pathway, and* (3) *Taking Steps Towards Action*. Themes are described below with illustrative quotes and also summarised in Table 2.

**Table 2:** Theme summary

Theme	Description	Findings	Key Questions from Interview Guides
*Navigating the role of Cancer Care Clinicians in the ECRP*	• Explores patient’s and CCC’s perceptions of their initial exercise conversations with their CCC, including the CCC’s role within the overall referral pathway.	Major Considerations • CCC exercise conversation promoted the credibility of exercise as adjunct cancer care treatment. • Perceived lack of awareness of EP services and their role in cancer care provision among patients. Additional Considerations • CCC exercise conversation was perceived as an effective cue to action. • Perceived sense of assumed exercise knowledge within patients	Patient • Did you find the brief consultation about exercise with your cancer care clinician helpful? • What information about exercise were you provided with during this consultation? • Were you satisfied with the amount of information provided to you during the consultation? CCC • Describe how your patient/s responded to including exercise as part of the information you discussed during the consultation? • How did your patient’s respond to the referral of an exercise physiologist?
*Implementing Patient-Orientated Care within a Standardised Pathway*	• Describes the multitude of ways the ERCP pathway promotes a patient-orientated approach within its design and implementation.	Major Considerations • CCCs tailored the content and timing of their exercise conversation based on their patient’s health profile and emotional state. • Patient orientated approach within the referral pathway was well-received by patients. Additional Considerations • Personalised approach in EP consultations extended to the tailoring of exercise recommendations including identification and referral to appropriate EP clinics.	Patient • What was discussed when the exercise physiologist contacted you? • What was the most helpful information you received from the exercise physiologist? CCC • How did you initiate the conversation about exercise with your patient/s? • At which consultation did you first initiate the conversation about exercise? EP • Can you describe (step by step) the approach you undertook with this trial? • What parts of the overall consult do you think were most effective? Least effective?
*Taking steps towards action*	• Identifies how the referral pathway aligns with a number behaviour change principles which may assist patients in taking steps to become more physically active.	Major Considerations • Referral pathway reduces barriers to accessing exercise counselling. • Increased patient knowledge of the Australian healthcare system including CDMPs sand rebated EP sessions. • Positive change in patients’ attitudes towards exercise. Additional Considerations. • Increased self-efficacy and perceived behavioural control amongst patients. • Referral pathway prompted initiation of exercise.	Patient • What was the most helpful information you received from the exercise physiologist? • What changes have you made to your exercise based on the recommendations or information you were given by the exercise physiologist? • Based on the consultation with your clinician and exercise physiologist, what are your initial thoughts about participating in exercise at this time?

### Navigating the role of cancer care clinicians in the ECRP

Most patients perceived the brief exercise conversations with their oncologists as an important motivator to exercise and that it provided credibility to the referral process itself. One patient highlighted: *“It was just reinforcing what I had thought myself anyhow. And to hear it [exercise advice] from the professional [CCC], it just makes you feel more comfortable with it”* (Patient 27, male). Another patient identified her consultation with her CCC as an effective cue to action: *“If she [CCC] hadn’t have mentioned it I probably would not have gone into this [consulted with the EP]”* (Patient 7, female). When asked to elaborate on the specific role of the CCC, some patients indicated that a CCC might be able to provide general exercise information (e.g., keep moving, increase walking, be as active as possible) however more specific information and advice (e.g. exercise prescription, inclusive of duration, intensity etc for a cancer patient) should come from an exercise specialist.

*I believe everyone should be doing their own jobs. She’s [CCC] not an exercise therapist, she’s an oncologist, so I believe the proper way was to see the exercise physiologist and, in accordance with my condition create a program. The oncologist looks after the chemo treatment and my progress but she’s not looking too much at my physical activity, it’s up to me and the [exercise] physiologist.* (Patient 12, male).

CCCs however perceived that the majority of patients were unaware of EPs or their important role in the overall cancer care and exercise pathways. Further, the CCCs perceived that the lack of EP knowledge seemed to go hand-in-hand with a sense of assumed knowledge about exercise in general.

*It made me laugh the number of patients who told me that they knew enough about exercise and they didn’t need to be referred on [to an EP] because they knew it all and I can tell you at their next follow up visit they still hadn’t done any exercise.* (CCC3, female).

As alluded to by all CCCs, this sense of assumed knowledge seemed to be quite common in many patients, and an influential factor in uptake of the EP referral offer.

### Implementing patient-orientated care within a standardised pathway

Throughout the ECRP, CCCs and the EP reported utilising a patient-orientated approach to their exercise conversations. Within the initial stages of the referral pathway, CCCs tailored the content and timing of their brief exercise discussion on a range of factors (e.g., type of cancer, symptoms, emotional state, information needs and exercise history).

*I find in the initial consultation there’s a lot to go through. When somebody has had 6 months of chemotherapy, they’re grappling with recovering from their surgery and there’s a lot of discussion specifically about cancer management and radiotherapy. I do talk about general lifestyles but it’s really not so much of a priority at that appointment…I just find those initial consultations very overwhelming for most patients.* (CCC2, female)

Similarly, the EP involved in the piloting of the ECRP emphasised that taking a personalised detailed approach in their consultations was beneficial to the tailoring of exercise recommendations:

*What I thought was most beneficial is to ask them their medical history first, so what cancer they were diagnosed with, what treatments they’ve had, where in the treatment journey they are, so I can understand where they are at. And then after I’ve got that information, I can then tailor my recommendations for them. For example, if they had breast cancer and they just had surgery then their upper body range of motion would be compromised or would be a bit lower so encouraging strength training and aerobic exercise is really helpful.* (EP, female)

Within EP consultations, the tailoring of exercise information also extended to identification and referral to an EP clinic outside of the study, allowing for convenient and enjoyable exercise opportunities to be identified:

*So not only was I giving them recommendations but also helping them out in finding what clinics are close to them, what they’re interested in, were they interested in one-on-one sessions or were they interested in group sessions with other cancer patients or survivors? So really tailoring that.* (EP, female)

This patient-orientated approach to exercise conversations within the referral pathway was well-received by the majority of patients. One patient highlighted his satisfaction in regard to receiving tailored suggestions for localised EP clinics:

*She [EP] asked me my address and where I lived and she looked up places where I could go and she said [local gym] had a good reputation and she thought that they’d do the job very well… She mentioned another in [local gym] but then she said she thought [local gym] would be better for me. It was more useful for me and more tailored to what I want as opposed to just going to a gym and hoping on a machine.* (Patient 27, male)

### Taking steps towards action

Throughout the referral pathway, stakeholders identified the incorporation of numerous behaviour change principles which influenced the perceived acceptability and feasibility of the model, including pathways for direct referral.

*It was definitely very easy for the patients because without that [referral from CCC], patients have to go to the GP themselves and go get that referral. It’s that extra step which could be a barrier for a lot of patients because they are already so busy with what they are going through and all their treatments, sometimes exercise could be the last thing they want to do or care about…* (EP, female).

In addition, the referral pathway provided opportunity to increase patient’s knowledge concerning the relationship between exercise and cancer as well as how to seek exercise support and tailored exercise services.

*The most helpful thing to me was, I would know what facilities I could contact for my situation if I needed to, that’s more useful than anything. I think I’m going to have a better resource, sort of a list to go to, probably more specific and more appropriate than I might’ve compiled on my own.* (Patient 9, male)

Patients also valued learning about the Chronic Disease Management Plan and rebated EP sessions offered through the Medicare System, many of which had no prior knowledge of and most intended to use these services in the future. Notably, participants linked this newly developed knowledge of EP services with a sense of empowerment and self-efficacy, and potentially perceived behavioural control: *“I feel more confident really that if I need to get further information, I can sort it out probably more readily now than I would’ve been able to before”* (Patient 9, male).

Many patients also explained a change in attitude towards exercise after participating in the study. Patient 31(female) summarised, highlighting a shift in their priorities: *“… I should be prioritising my health, but because I am a Mum and I work, other things are killing my time. But after speaking with the EP, it hit me, I should be doing this [exercising regularly], I have to do this*!” Some also outlined that this had already transferred into action:

*What I’ve changed is my mindset. When I come home from my appointment and I’m exhausted I really just feel like sitting down and doing nothing but I sort of push myself for a couple of hours more doing stuff to keep me active … By the time I finish I’m really, really exhausted and then I sleep better.* (Patient 10, female)

## Discussion

Our findings support the feasibility and acceptability of the proposed ECRP model as an initial approach for improving oncology-patient exercise communication and referral pathways. Moreover, these findings also provide areas of refinements, modifications and enhancements for further progressing the ‘next steps’ of the ECRP model. In sum, this includes a patient-orientated approach across all stages of the model and the incorporation of established behaviour change techniques to support the initiation of exercise including, building self-efficacy, reducing perceived barriers, emphasising health benefits, changing attitudes and offering a cue to action.

Supported by previous research [8, 9, 14], a brief communication exchange between oncologists and their patients about exercise should be an essential component of the oncology consultation and was included as the initial stage of the ECRP. Exercise communication can be delivered by any member of the oncology care team (e.g., nurse) [4] however an oncologist’s recommendation in the first instance may be particularly valuable [29, 30]. Patient participants in our study and in other literature found timely exchange with CCCs can be reassuring and motivational, particularly because of the level of trust patients have with their oncologist [14, 30]. Oncologists and physicians often serve as central gatekeepers in providing health care information [31], and are recognised as highly credible sources to provide patients with accurate and reliable information about cancer care, including exercise [32, 33]. Despite the importance of having a brief exercise communication between patient and oncologist, our findings indicate potential friction related to patient and CCC perceptions of EPs and the role they play within the referral pathway. As part of the brief oncology-patient communication exchange, CCCs need to clearly outline the overall referral process and each health professionals’ responsibilities within it to enhance uptake. This should involve CCC’s acknowledging their own limitations regarding exercise prescription and explaining to patients why EPs are the most appropriate sources of information and support.

Patient-oriented care was a strong and consistent theme shared by all participants (i.e., patients, oncologist, EP) throughout this study, and is further supported within exercise oncology research [34]. A particular emphasis was placed on offering tailored exercise oncology advice and prescription from an EP, further highlighting the need to move away from a ‘one size fits all’ mindset [35]. The EP in this study went beyond traditional tailoring, and provided information concerning local exercise services/programs for cancer patients (including free, subsidized and/or full paid programs and services), local facilities that could accommodate (e.g., proper equipment, supervision, etc.) cancer patients, and local EPs or health professional with cancer and exercise knowledge. This level of tailoring not only provides patients with access to cancer specific exercise resources and services, it also provides a move towards self-management via community-based services. This transition is essential and effective for improving exercise confidence, self-efficacy and in turn supporting long-term lifestyle behaviour change [36, 37].

Cancer specific knowledge to tailor exercise to the complexities of each type of cancer and individual patient factors (e.g., personal, environment, and social determinants) is crucial to providing the most effective, patient-oriented care and was critical in the referral pathway. Cancer is a complex and everchanging disease that includes hundreds of different types, stages, treatments, and side effects. This level of cancer specific knowledge aided the EP in providing tailored advice which was important to patients, and as previously indicated can have a positive impact on patient confidence and exercise behaviour change [36, 37]. Having access to training and education specific to exercise oncology continues to grow throughout the field [22]. In Australia, we have seen a further progression where tertiary (e.g., Graduate Certificate of Exercise Medicine Oncology at Edith Cowan University) and professional (e.g., EX-MED Cancer Professional Development Course, https://www.exmedcancer.org.au/) courses are being offered to EPs interested in working with people living with cancer.

The findings from this feasibility study, demonstrates the potential viability of the ERCP pathway, however, broad policy and practical changes are warranted to make the pathway feasible for real-world implementation. In terms of policy, a systems level change to the Medicare Care Plans is essential as it currently is a barrier for patients, oncologist, and EPs. As identified earlier, in Australia only GPs can establish and refer their patients with cancer to a Medicare Care Plan, enabling them to receive five subsidised EP sessions. This current referral process does not allow an oncologist to directly refer patients to an EP or exercise program. Providing oncologists with the capacity to provide a direct referral to an EP and/or exercise program would not only address patient barriers, but also improves the effectiveness, efficiency, and coordination of services within the referral pathway enhancing the overall quality of cancer care [38].

Our study also addressed a practical issue commonly reported by exercise practitioners working within the cancer field. In a number of referral pathways, the patient is tasked with accessing the EP to initiate exercise advice and/or access to services and programs [39, 40]. This adds a further obstacle for patients, and in many instances results in patients never contacting an EP or engaging in exercise programs/services [12]. Our study shifted the focus, where the oncologist (with consent) shared relevant patient information with a nominated EP and the EP then contacted the patient. Having the EP make initial contact was well received by patients, many indicating that this was a crucial element in ‘getting the process’ started. To further assist in reducing access related barriers, this EP initiated approach could be combined with mixed modes of service delivery (i.e., telehealth and face to face). Specifically, telehealth appointments could be used to review patient progress and assist in exercise maintenance in between physical visits.

The policy changes regarding the Medicare Care Plans and practical changes in terms of EP access have potential to improve the exercise communication and referral pathway however, they also come with some challenges to health service systems. Most obvious is the funding pressures and inadequate resources that continues to plague global health systems [10, 23] and the roll-on effect this has on services that may be deemed to be adjunct or supplemental, such as exercise. This is where the cultural shift becomes critical, ensuring that all levels of the health system (i.e., organisational management, oncology workforce, health practitioners, and patients) are committed to embedding exercise as part of standard care. Integrating an EP service and an exercise facility onsite at all hospitals would be an essential step in building a culture where exercise counselling is viewed as a vital part of a cancer patient’s treatment via increased awareness and accessibility of services and a coordinated care approach.

This study provided rich, in-depth information from patients, oncologists and an EP, all of which are critical players in understanding how to best integrate exercise into standard cancer care. To our knowledge this is the first to test a practical exercise communication and referral pathway in a real-world setting, exploring innovative ways to better implement exercise and EPs into standard care to reach more people living with cancer. Although the study provided rich data from both patients and clinicians/practitioners, the subgroup of CCCs/practitioners was small, all were employed by the same public hospital (except for the EP), and were located in an urban centre. Thus, findings may not translate across all cancer care centres or other regions, particularly rural and remote areas. Moreover, the CCCs were known to the research team and thus may have had a more proactive perspective regarding the project compared to other CCCs, further limiting generalisability. In addition, the lack of diversity in the patient cohort (i.e. nearly 50% breast cancer) and inclusion of only one EP’s perspective also limits generalisability and transferability.

Further, the purpose of this study was to explore changes to an exercise communication and referral pathway, with particular consideration of the implementation process. As such, once the EP completed the initial discussion with the patient the intended aim was reached and no further data was collected. Therefore, it was not possible to determine if the ECRP resulted in a change to exercise engagement or access to exercise services/programs. Further experimental research (e.g., RCT, pre-post trials) on a diverse (e.g., socio-geographic) sample is needed to examine effectiveness of ECRP at increasing exercise. An additional limitation includes the study’s attrition rate. Approximately 40% of patients (N = 14) who consulted with the EP dropped out of the research study prior to completing their interview. As such, attrition bias may be present within the sample. However, given the target population, it is important to note N = 3 of these participants did not complete interviews because of death or significant illness.

## Conclusions

In sum, the piloted ECRP is highly acceptable in its’ current form, with only minor improvements to the structure and processes of the model suggested. Specifically, CCCs need to provide a more comprehensive overview of the referral process and the roles and responsibilities of all stakeholders involved in it. However, for this referral pathway to be more feasible in the future, a number of practical (i.e., formal integration of CCCs and exercise specialists, EP initiated contact with patients) and policy changes (i.e., changes to Medicare, including direct oncologist referral) need to occur. Collectively, these changes may make a significant contribution to improving access related barriers to cancer care services and enhancing the health and wellbeing of cancer patients.

### Electronic supplementary material

Below is the link to the electronic supplementary material.

Supplementary Material 1

Supplementary Material 2

## Data Availability

The datasets generated during and/or analysed during the current study are available from the corresponding author on reasonable request.
