# Supplementary figures and images for: Exploring the practicality and acceptability of a brief exercise communication and clinician referral pathway in cancer care: a feasibility study
Source: BMC Health Serv Res. 2023 Sep 22;23:1023. doi: 10.1186/s12913-023-10003-x (PMC10517509; doi:10.1186/s12913-023-10003-x)

**Additional File 2.** Exercise referral pathway for cancer patients

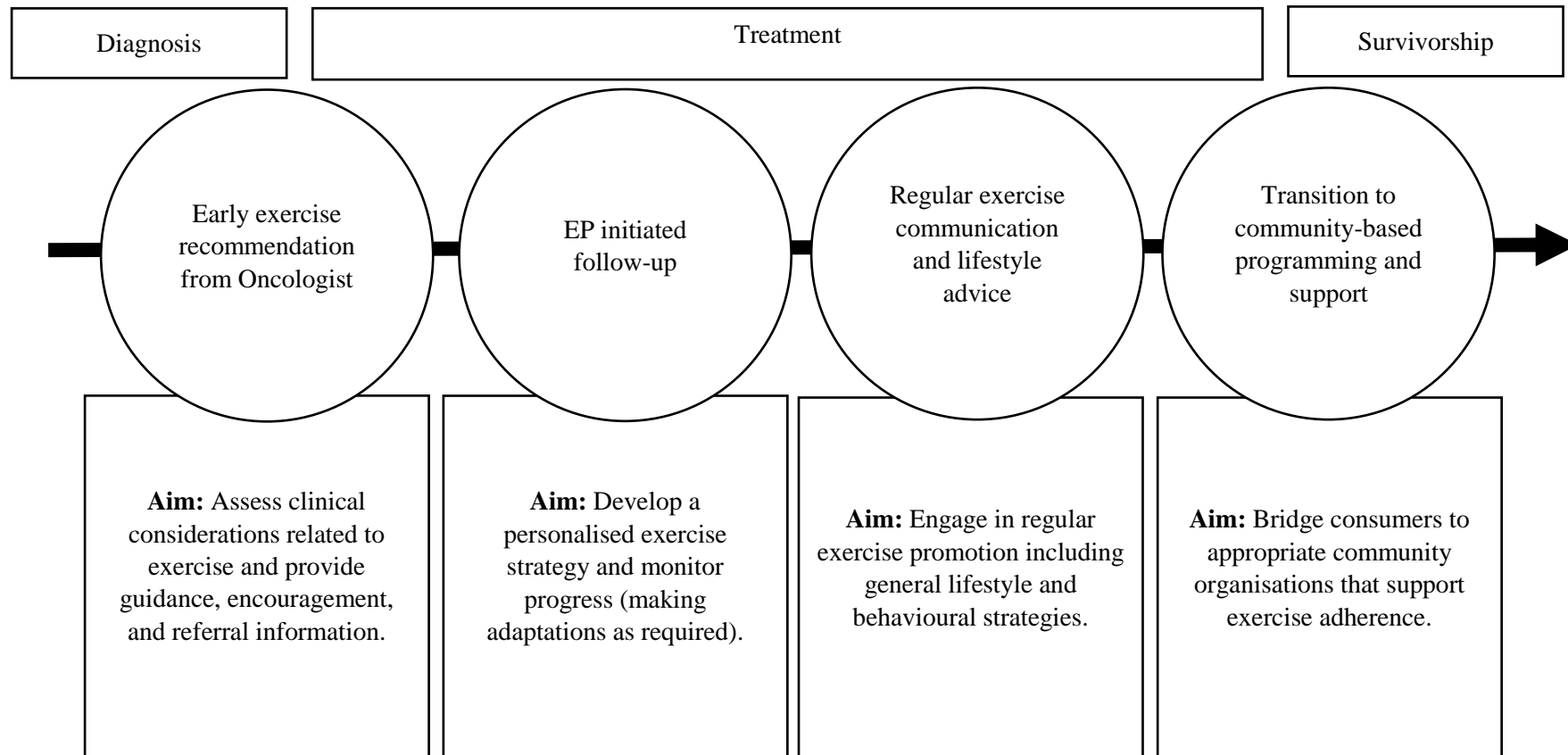

Supplement: Supplementary file 1 — Supplementary Material 1 [file 12913_2023_10003_MOESM1_ESM.pdf]
