# Supplementary material for: Exploring the practicality and acceptability of a brief exercise communication and clinician referral pathway in cancer care: a feasibility study
Source: BMC Health Serv Res. 2023 Sep 22;23:1023. doi: 10.1186/s12913-023-10003-x (PMC10517509; doi:10.1186/s12913-023-10003-x)
Supplement: Supplementary file 2 — Supplementary Material 2 [file 12913_2023_10003_MOESM2_ESM.pdf]

Additional File 3 – Participant Question Guides

Patients

1) What were your initial thoughts about exercise during your cancer treatment and beyond (before you spoke to your Cancer Care clinician or the exercise Physiologist Lauren)?
    a. *Do you think it is important? Why? Why not?*
    b. *Do you think you should be doing it? Are there reasons why you should not be performing exercise during or after your treatment?*

2) Did you find the brief consultation about exercise with your cancer care clinician helpful?
    a. *What was most helpful? What was least helpful?*

3) What information about exercise were you provided with during this consultation?
    a. *Recommendations? Benefits? Cancer specific effects? Exercise prescriptions? Information about other resources? Hard copy resources or web-based resources? Anything else?*

4) Were you satisfied with the amount of information provided to you during the consultation?
    a. *If yes, what information in particular was satisfactory?*
    b. *If not, can you describe what additional information about exercise do you think should be discussed during this time?*
    c. *Should more time be spent on this discussion?*

5) At which consultation was exercise discussed with you? When is the best time to receive this information?
    a. *e.g. before, during or after cancer treatment?*

6) Did your cancer care clinician (oncologist) talk to you about other people who could help with initiating an exercise program for you? If so, who did they suggest?
    a. *Nurse? Physiotherapist? Exercise physiologist? Personal fitness trainer? Friends or family?*

7) How useful was the cancer and exercise resource page (i.e., organgy-red exercise sheet) that you received when you signed up for this study?
    a. *What parts were most helpful?*
    b. *What recommendations do you have to improve the resource page?*

8) Can you share with me how you felt about having an exercise physiologist contact you? Was this an effective way to start the referral to exercise pathway?
    a. *What barriers or challenges did you encounter during the exercise referral procedure? Logistical?*
    b. *Can you suggest any alternative procedures that may be more effective?*

9) What was discussed when the exercise physiologist contacted you?
    a. *Exercise opportunities? Access? Funding? GP referral?*

    b. *Benefits of exercise? Types of exercises most appropriate for you?*
    c. *Exercise recommendations?*

10) What was the most helpful information you received from the exercise physiologist?
    a. *Is there any other information that you would like?*

11) What changes have you made to your exercise based on the recommendations or information you were given by the exercise physiologist?
    a. *Did you obtain a CDM plan from your GP to access the services from an exercise physiologist?*
    b. *Did you contact or book in a consultation with an exercise clinic? If so, can you describe the process of this? What would you change to make the process easier?*

12) Based on the consultation with you clinician and exercise physiologist, what are your initial thoughts about participating in exercise at this time?
    a. *Are you more likely to engage in exercise right now? If so, are there any factors that you need assistance with in getting started?*
    b. *If not why, can you explain what might be holding you back from participating in exercise?*

13) Is there anything else you would like to share with us concerning this experience?

<u>Oncologist</u>

Part 1 – Experiences with resources and referral process

1. How did you initiate the conversation about exercise with your patient/s?
    a. *Was this effective? How did they respond?*

2. At which consultation did you first initiate the conversation about exercise? Initial, 2nd consultation or after a few consultations?
    a. *When is the best time to initiate conversations about exercise? Post-diagnosis, during treatment or remission. Why?*
    b. Approximately how much time was spent talking about exercise during the consultation? *Was that enough time to comprehensively discuss exercise guidelines/benefits/recommendations or was more needed?*

3. What information did you include/exclude in your conversation about exercise? Why?
    a. *Potential risks*
    b. *Cancer specific benefits*
    c. *Mental wellbeing*
    d. *Potential support people other than exercise physiologist*
    e. *Physical activity guidelines and recommendations*

4. How did you use the guiding information/resource (i.e., the blue 3As sheet)?
    a. *Did the resource help guide your discussion about exercise with the patients?*
    b. *What did you like about the resource? Why?*
    c. *What would make the resource more useful?*
    d. *What information do you require to further support this discussion?*
    e. *Other resources (e.g., training, workshop, etc.)?*

5. How confident are you in discussing (Why/why not?):
    a. *Exercise guidelines*
    b. *Benefits of physical activity*
    c. *Recommendations for patients to be physically active*
    d. *Is there any additional assistance which could be provided which would increase your confidence discussing these topics (e.g., online learning, workshops or additional suggested readings)?*

Part 2 - Perceptions of patient responses

6. Describe how your patient/s responded to including exercise as part of the information you discussed during the consultation?
    a. *Positive, negative or indifferent? Were they open to discussing options?*

7. What questions or concerns did your patients indicate for exercising during/after cancer treatment?
    a. *No questions? Why exercise? What type and how much is needed to get the benefits? Is it safe for me to exercise?*

8. How did your patient's respond to the referral of an exercise physiologist?
    a. *Did they know what an exercise physiologist is?*

   b. *How receptive were patients when you told them that an EP would contact them to discuss exercise?*
   c. *What questions/comments did they have about the exercise physiologist?*

9. Can you describe any other barriers that you believe to impact your patients engagement and participation in exercise during the referral process?
   a. *Access and opportunity? Medicare funding structure?*
   b. *Clinician based barriers or challenges (e.g., knowledge, expertise, time, etc.)*
   c. *Health status/comorbidities*

10. Other than the referral process you tested during this feasibility trial, do you have any other suggestions that may assist with implementing exercise for your patients and improve engagement and participation of exercise for oncology patients?

<u>Exercise Physiologist (EP)</u>

Part 1 - Description of the process

1. *How would you describe the role of the EP (or exercise specialists) in thereferral pathway?*
    a. *Assess physical activity levels*
    b. *Build awareness of PA guidelines and recommendations*
    c. *Prescribe tailored exercise*
    d. *Educate patients on cancer specific benefits*
    e. *Motivate patients to become more active*

2. Can you describe (step by step) the approach you undertook with this trial
    a. *What are your thoughts about this approach? How does it enhance the current referral process of…?*
    b. *As an EP, do you think more can be done to ensure that the EP is part of this process? Explain*

Part 2 - Understanding more about the process

1) What are your thoughts about the EP contacting patients via telephone?
    a. *Was this effective in reaching patients?*
    b. *Can you describe the logistics of this approach. How was the process initiated? What details were you given to assist in initiating contact with the patient?*
    c. *Is this practical for future use (OR how could this process be adapted to work in other hospitals?)*
    d. *What are your 'lessons learned' from being involved in the process?*
    e. *Did the patients indicate anything about this? Were they positive, negative, indifferent about being contacted by the EP?*

2) What main questions or concerns did your patients have during the tele-consult?
    a. *No questions? Why exercise? What type and how much is needed to get the benefits? Is it safe for me to exercise? Programming? Resources? What PA opportunities are local to me?*
    b. *Something other than exercise? How do I access Medicare funded sessions? How many free sessions do I receive?*

3) Were there specific guiding information/resources that you used during the consult?
    a. *What information/resources do you require to further support this consult?*

4) What parts of the overall consult do you think were most effective? Least effective?

5) In terms of the referral process tested in this trial, do you have any other suggestions for refining this process to better assist with improving engagement and participation of exercise for oncology patients?

6) What do you think are the important next steps, following this referral process, to improving engagement and participation of exercise for oncology patients?
    a. *Policy change? Access, opportunities, resources?*
    b. *What is the role of the EP (or exercise specialist) in this?*
